# Factors Influencing Public Attitudes towards COVID-19 Vaccination: A Scoping Review Informed by the Socio-Ecological Model

**DOI:** 10.3390/vaccines9060548

**Published:** 2021-05-24

**Authors:** Ghadir Fakhri Al-Jayyousi, Mohamed Abdelhady Mabrouk Sherbash, Lamees Abdullah Mohammed Ali, Asmaa El-Heneidy, Nour Waleed Zuhair Alhussaini, Manar Elsheikh Abdelrahman Elhassan, Maisa Ayman Nazzal

**Affiliations:** 1 Department of Public Health, College of Health Sciences, QU Health, Qatar University, Doha P.O. Box 2713, Qatar; msherbash@qu.edu.qa (M.A.M.S.); la1605823@qu.edu.qa (L.A.M.A.); nwaleed@qu.edu.qa (N.W.Z.A.); melhassan@qu.edu.qa (M.E.A.E.); 2 School of Medicine and Dentistry and Menzies Health Institute Queensland, Griffith University, Gold Coast Campus, Southport, QLD 4222, Australia; a.el-heneidy@griffith.edu.au; 3 Department of Pharmacy, Faculty of Medicine and Health Sciences, An Najah National University, Nablus 44839, West Bank, Palestine; s11525766@stu.najah.edu

**Keywords:** COVID-19, vaccine, hesitancy, acceptance, refusal, willingness, ecological model, scoping review

## Abstract

Major hindrances to getting a COVID-19 vaccine include vaccine hesitancy, skepticism, refusal, and anti-vaccine movements. Several studies have been conducted on attitudes of the public towards COVID-19 vaccines and the potential influencing factors. The purpose of this scoping review is to summarize the data available on the various factors influencing public attitudes towards COVID-19 vaccination. This scoping review was conducted according to the Preferred Reporting Items for Systematic Reviews and Meta-Analyses extension for Scoping Reviews (PRISMA-ScR) Statement. PubMed, Embase, Web of Science, and Cochrane Central were searched without restrictions to reclaim all publications on the factors that shape individuals’ attitudes towards COVID-19 vaccines from 1 January 2020 to 15 February 2021. Fifty studies were included. The scoping review revealed that the factors influencing public attitudes towards COVID-19 vaccines were embedded within the different levels of the socio-ecological model. These factors included the sociodemographic characteristics of the individuals, individual factors, social and organizational factors. In addition, certain characteristics of COVID-19 vaccines themselves influenced public attitudes towards accepting the vaccines. Understanding various population needs and the factors shaping public attitudes towards the vaccines would support planning for evidence-based multilevel interventions in order to enhance global vaccine uptake.

## 1. Introduction

Coronavirus disease 2019 (COVID-19) is a contagious and pathogenic viral infection caused by severe acute respiratory syndrome coronavirus 2 (SARS-CoV-2), a specific type of coronavirus that was first discovered in Wuhan, China [1]. It was declared a global pandemic by the World Health Organization on the 11 March 2020. The pandemic caused by COVID-19 has infected more than 125 million people and killed at least 2.5 million globally and is becoming a leading cause of death [2]. This virus has become a major concern around the globe, having so many consequences on the healthcare system and economy and instilling fear in communities [3,4]. The main mode of transmission is through droplets, direct contact with infected patients; it can also be transmitted through fomites, by touching contaminated surfaces or objects [5]. People who are at increased risk of getting severe infection include the elderly and those who have chronic diseases [6].

Although many efforts have been dedicated to the implementation of suppression strategies including travel bans, partial/full lockdown, contact tracing, and social distancing, the transmission of the virus is more likely to rebound when these strategies are lifted [7]. Consequently, for a long-term approach to combating this epidemic, the development and use of vaccines is essential [8].

Vaccination stimulates the immune system to develop antibodies to fight a specific infectious agent in the body [9]. They have been used to eliminate and significantly decrease morbidity and mortality associated with different infectious diseases [10] by providing benefit to those who get vaccinated and also protecting communities through reducing transmission of the disease [10]. Via herd immunity, a high uptake of COVID-19 vaccines can also help protect people who cannot get a vaccine such as those with compromised immune systems and young children [11]. Getting efficacious results from a vaccine does not solely rely on accessibility/uptake, but also depends upon the public’s acceptance and willingness to get vaccinated [11]. Other major hindrances to getting a vaccine include vaccine hesitancy, skepticism, refusal, and anti-vaccine movements [12]. In 2019, vaccine hesitancy was identified as one of the ten challenges to global health [13], and this concern has grown throughout the COVID-19 pandemic [14]. While prior studies looked at predictors of vaccine acceptance and uptake, it is worth noting that emergency-released vaccines differ from established vaccinations in many aspects [15], and newer vaccines are usually met with greater skepticism [16].

Several studies have been conducted on attitudes of the public towards COVID-19 vaccines and potential influencing factors [15,17]. It is imperative to investigate the different factors influencing attitudes and perceptions of people related to COVID-19 vaccines. Vaccine refusal has a variety of causes which differ depending on regional, cultural, and social factors [18]. Understanding different vaccine attitudes is particularly significant as diverse vaccine refusal strategies that address the needs of different groups can be developed [19]. In the light of the COVID-19 pandemic, there is an urgent need for a more comprehensive and detailed understanding of attitudes toward vaccines and the factors affecting vaccine intention in order to adjust public health messages as appropriate [20]. Therefore, in this scoping review, we sought to rapidly explore the determinants influencing public attitudes with respect to COVID-19 vaccines and provide a more comprehensive and nuanced understanding of how these factors shape certain perspectives and behaviors.

## 2. Materials and Methods

This scoping review was conducted according to the Preferred Reporting Items for Systematic Reviews and Meta-Analyses extension for Scoping Reviews (PRISMA-ScR) Statement (see Appendix A) [21]. The study has no written or published a priori protocol. Our research question was as follows: what are the factors influencing public attitudes with respect to COVID-19 vaccines? After identification of the research question, we identified relevant studies, selected the studies, charted the data, and collated, summarized, and reported the findings.

### 2.1. Information Sources

PubMed (National Library of Medicine, Bethesda, MD, USA), Embase (Elsevier, Amsterdam, The Netherlands), Web of Science (Clarivate Analytics, Philadelphia, PA, USA), and Cochrane Central (Cochrane, London, UK) were searched without restrictions to reclaim all publications on the individual factors, sociocultural factors, and environmental factors that shape an individual’s decision (attitude) towards COVID-19 vaccines from 1 January 2020 to 15 February 2021. Table 1 describes the search strategies used to collect published articles from the databases. Reference lists of the selected articles were also searched for articles that might have been missed in the online database search.

### 2.2. Eligibility Criteria

In this scoping review, all the articles published between 1 January 2020 to 15 February 2021 about the factors that shape public attitudes towards COVID-19 vaccines were included. The articles selected were only in English, with human subjects aged 16 years and above, excluding healthcare workers who are obligated to get a vaccine. Articles were only included in English since it was the major language of the available articles in the databases at the time this search was conducted.

Two authors independently screened abstracts and citations retrieved from the search and each author (out of the seven authors) was given a specific number of articles to assess full texts of the relevant records to be included in the review. When dealing with duplicates, the most recent version of the article with the largest sample size was included. During the study selection and assessment process, the first author was responsible for resolving any disagreements and final evaluation.

### 2.3. Data Charting Process

The relevant data were abstracted from the eligible articles in pre-structured data charting forms (Excel (Microsoft Corporation, Redmond, WA, USA) and Word (Microsoft Corporation, Redmond, WA, USA) documents). The following information was included: the first author of the study, publication year, study design, population, study setting, mean age of the participants (in years), sample size, attitude towards COVID-19 vaccines, and the various factors shaping these public attitudes. Descriptive statistics (percentages) were reported for some studies to reflect the rate of positive or negative attitudes towards the vaccines. The final column in the table reported percentages to reflect prevalence of the factors shaping the attitudes, and for some studies, the *p*-value was reported to reflect the significant association between the factors and public attitudes towards the vaccines.

### 2.4. Synthesis of Results

Description of the scope of literature was presented in tables according to the key levels of the socio-ecological model [22] which showed how a health determinant (public attitudes towards COVID-19 vaccines) can be influenced by the various factors embedded in different levels. In our review, we summarized and clustered the factors that influenced public attitudes towards COVID-19 vaccines into sociodemographic characteristics, individual factors, and social and organizational factors. The final set of factors that were related to the specific characteristics of COVID-19 vaccines were summarized and reported in a separate table.

### 2.5. Data Analysis

Outcome data related to the factors that influence public attitudes towards COVID-19 vaccines were summarized and clustered into the different levels of the socio-ecological model. Meta-analysis was not performed due to heterogeneity in the contextual and environmental factors including the healthcare system in the countries of the selected studies in our review.

## 3. Results

**Figure 1:**
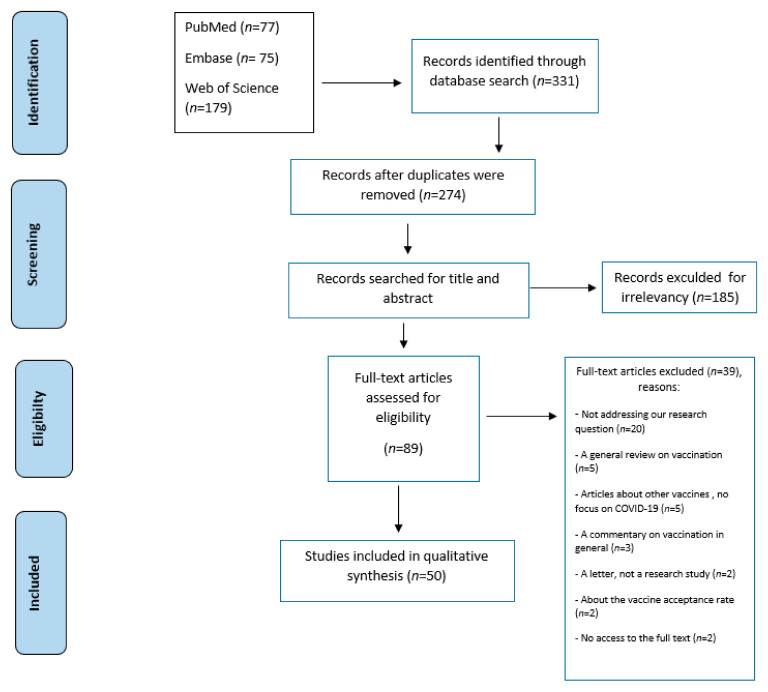
PRISMA flow diagram of study selection.

In total, 331 records were retrieved from the electronic database search. The remaining records, after removing the duplicates, amounted to 274 records. After screening the titles and abstracts, 185 were excluded; the remaining 89 full-text articles were assessed for eligibility, and 50 studies were reserved for this review. The PRISMA diagram illustrates the study selection process and shows the reasons for exclusion for other studies (Figure 1).

### 3.1. Study Characteristics

The studies were divided by design into one experimental study [23], 39 cross-sectional studies [24,25,26,27,28,29,30,31,32,33,34,35,36,37,38,39,40,41,42,43,44,45,46,47,48,49,50,51,52,53,54,55,56,57,58,59,60,61,62], five literature reviews [63,64,65,66,67], one systematic review [68], one randomized controlled trial [69], one longitudinal survey with two experiments [70], one Gallup panel [71], one media analysis study through a proposed novel behavioral dynamics model SRS/I (susceptible–reading–susceptible/immune) for the microblogging platform Weibo on social media [72], and one conference paper [73]. The majority of these cross-sectional studies reported their findings from one country, one study reported data from 19 countries [25], another study conducted legal analysis for several countries [40], and others reported findings from two or three countries from Europe, America, Canada, and the Middle East [29,49,59]. The sample size of the reviewed studies ranged from 101 participants in an RCT study [69] to 13,426 participants in a survey conducted in 19 countries [25].

An online (web-based) survey was the most common data collection method applied in the cross-sectional studies in our review, of which:

− Some studies reported using social media platforms including Facebook, Twitter, and LinkedIn to collect data [26,38,41,62,72];
− Some studies reported using specific platforms to upload surveys including crowdsourcing platform Amazon Mechanical Turk (mTurk, Amazon, Seattle, WA, USA) [32,61], computer-assisted web interviewing (CAWI) and computer-assisted telephone interviewing (CATI) [34,53], and Qualtrics (Seattle, WA, USA) [47,55];
− One study used an online questionnaire to examine public attitudes towards COVID-19 vaccines in three phases: pre-lockdown, during lockdown, and post lockdown with different participants in every phase [41], and another study conducted two-wave follow-up online surveys [55];
− One study used an online survey with a semi-structured questionnaire following the snowball sampling technique [60];
− One study used open-ended questions to ask about the factors influencing attitudes towards vaccines [47];
− One study used an Internet survey and telephone interviews [35];
− One study used a face-to-face-administered questionnaire [37].

The target populations in the majority of the reviewed articles were adults from the general public. The youngest participants were recruited in a study from Italy in which the age ranged from 15 to 85 years [53], and another study was conducted among parents and guardians aged 16 years and above who reported living in England with a child aged 18 months or under [26]. Only three studies were conducted among populations with specific demographics such as the working population in Hong Kong (HK), China [52], people with respiratory chronic diseases and older adults aged 65 and above [47,55], and the black American community in an RCT study [69].

The reported vaccine acceptance rate in the reviewed articles ranged from 29.4% to 86% [23,24,26,45,46,47,48,49,51,72]. A study from the Middle East [49] reported a low rate of acceptance among the public from Jordan, Kuwait, and Saudi Arabia (29.4%), while a higher acceptance of 53.1% was reported in a study from Kuwait [48]. On the other hand, 86% of people in the UK expressed their willingness to receive a vaccine [47]. The information on the baseline characteristics of the selected studies can be found in Table 2.

### 3.2. Sociodemographic Characteristics Shaping Public Attitudes towards COVID-19 Vaccines

Twenty-eight studies depicted the sociodemographic factors associated with public attitudes (Table 3). Age [23,25,27,29,31,34,35,36,38,51,52,53,63,65,73], educational level [23,24,25,27,30,35,36,42,47,49,50,51,63,65,68,71,73], gender [24,25,28,29,30,31,32,34,38,48,49,51,52,65,68], race [26,29,35,36,47,51,63,68,71,73], and income status [24,25,26,29,47,50,51,63,65,68] were the most common factors reported. White individuals older than 25 years who have a high education level and high-income status were more likely to report positive attitudes towards the vaccines. The majority of the reviewed studies reported that men were more willing to accept a vaccine than women [28,29,30,38,48,49]. Only one study in the USA reported higher willingness among men [32].

Moreover, other factors were included, such as health condition, people with chronic diseases [29,31,49,65,73], occupation status [27,30,46,50,53,73], marital status [27,28,52], place of residence [29,51,68,73], women being pregnant [29] or having children [30], and those who have health insurance or not [30,31,36,51]. The reviewed articles showed that individuals who had chronic diseases, were employed, married with children, and had health insurance were more likely to report acceptance of COVID-19 vaccines.

### 3.3. Individual Factors Shaping Public Attitudes towards COVID-19 Vaccines

Several individual factors influencing public attitudes were reported in thirty-six studies (Table 4). Personal beliefs with regard to vaccines and COVID-19 [30,32,34,35,49,55,56,57,62,66,68,70], health literacy [33,65], knowledge [34,37,57,68], lack of trust in governments and companies producing the vaccines [25,35,37,39,59], perceived susceptibility and risk perception towards COVID-19 and side effects of the vaccines [24,28,36,38,45,46,47,48,56], social [61,70], religious [37], and political views [29,32,54,61,71], level of anxiety of getting infected [30], fear [24,30,37,38] and worries [39,59], confidence in academic institutions and producing companies [24,60], preference towards natural immunity [40], previous experience with flu vaccines or other vaccines [28,30,52,56,58], likelihood of infection and severity of the disease [41,43,48,58] were all considered as individual factors in this scoping review.

Our review showed that the individuals who believe that coronavirus is contagious and lethal, have good knowledge and score high on health literacy, are stressed, worried, and anxious about getting infected, trust the healthcare system, the government, and the companies producing the vaccines, and have positive experience with previous vaccines were more likely to accept COVID-19 vaccines than others. On the other hand, the individuals with greater conspiracy beliefs and political conservatism, and those with personal reasons to refuse a vaccine, including religious conviction, were hesitant and reported negative attitudes towards COVID-19 vaccines.

### 3.4. Social and Organizational Factors Shaping Public Attitudes towards COVID-19 Vaccines

Table 5 represents the articles that discussed the role of family, friends, healthcare providers, and employers in shaping public attitudes. In addition, it depicts the articles discussing the role of traditional (classic) and social media. Social networks and organizational factors that affect the attitude toward COVID-19 vaccines were reported in 10 studies. Social service and healthcare providers and the physician’s recommendation of vaccination [28,43,44,69] were the most prevalent ones. The individuals who were advised by a physician or any other healthcare provider to take a vaccine were more likely to have a positive attitude towards it than those who did not get any advice. The employer’s recommendation might also influence an individual’s attitude positively [25].

The influence of traditional media [42], type of messages received, and disinformation through the Internet and social media [23,37,64] were among the reported factors. The reviewed articles showed that misleading information shared on social media platforms would make individuals hesitant to take the vaccine. In addition, 78% of the participants in one study stated that their decision to get a vaccine was supported by their family and friends [31], especially when someone of their family members or friends was vaccinated [44].

### 3.5. Characteristics of COVID-19 Vaccines and Public Concern

Table 6 summarizes the literature that shows how some vaccine characteristics affect public attitudes. Efficacy [28,40,43,45,46,63,65,68,73], safety [25,28,35,47,63,65,68,73], cost [46,63,68,72], and adverse effects or toxicity of the vaccine [37,40,46,47,63,68,72,73] were among the vaccine characteristics that were reported to shape public concerns about COVID-19 vaccines. The reviewed articles showed that the individuals who had a negative perception towards vaccine efficacy, safety, and side effects would report unwillingness and hesitancy towards taking a vaccine.

Furthermore, immunity duration [68], timeframe of vaccination [63], fake or low-quality vaccines [46], country of vaccine origin [24,68] and information about inactivated vaccines [72] were other public concerns in the selected studies. The individuals who believed that immunity boosted by a vaccine would be for a short period, vaccine development was expedited, and the production process was pushed, and that the vaccines are most probably fake would report negative attitude towards COVID-19 vaccines.

## 4. Discussion

This scoping review of 50 articles systematically maps evidence on the influencing factors that may lead to COVID-19 vaccine hesitancy worldwide. Vaccine hesitancy and anti-vaccination movements represent an old phenomenon that threatens global health [74,75,76,77]. With the current situation of the COVID-19 pandemic, this can be a stumbling block in the global efforts to control the disease and its devastating consequences.

Vaccination is considered a vital element for public health; it is the most effective intervention for the primary prevention of communicable diseases. To enhance acceptance and uptake of vaccines, it is crucial to gain insight into the common factors that influence an individual’s decision-making process to help inform policymakers to develop effective strategies. This scoping review updates the latest information on the determinants that impact COVID-19 vaccine uptake. The review included studies that reported global data which offer an insight on how public attitudes towards COVID-19 vaccination varies around the world. It also demonstrated that these attitudes are influenced by a wide range of factors on multiple levels of the socio-ecological model. Sizeable evidence showed that sociodemographic factors such as age, gender, and income status, individual factors such as personal beliefs and risk perception, and social and organizational factors such as the role of significant others are among the most related determinants. In addition, certain characteristics of the COVID-19 vaccines themselves like efficacy, safety, and side effects influence public attitudes (Table S1).

The individuals’ attitudes towards the COVID-19 vaccination varied among the studies, with acceptance ranging from 29.4% to 86%. This discrepancy could be attributed to variations in the study population. Sallam et al. [49] reported a very low rate of acceptance among people in three Arab countries, Jordan, Kuwait, and Saudi Arabia (29.4%), as compared to a relatively higher acceptance of 53.1% reported in one study in Kuwait [48]. In contrast, as high as 86% of people in UK (mainly elderly and middle-aged at-risk adults) expressed their willingness to receive a vaccine [47]. This is in line with the findings of a recent systematic review [77] that showed a global variation in the rate of vaccine acceptance, with the Middle East being among the regions having the lowest rates. The review related this finding to the widespread embrace of conspiratorial beliefs in the region, which subsequently resulted in negative attitudes towards vaccination. This negative attitude in the Arab region is alarming.

In this review, 28 studies depicted the sociodemographic factors associated with public attitudes toward the COVID-19 vaccination. Coherent to findings from the literature [78], the most common factors found to influence vaccine acceptance at the microlevel were age, educational level, gender, race, and income status [23,24,25,26,27,28,29,30,31,32,33,34,35,36,44,45,46,47,48,49,50,51,52,53,63,65,68,71,72,73]. More willingness to receive a vaccine was reported in the older age group [23,25,27,31,34,35,38,53,65], while resistance, hesitancy, and lack of intention to be vaccinated emerged in the younger age group [29,35,51]. This could be attributed to differences in age distribution between countries, literacy level, and the fact that older adults are at a higher risk of morbidity and mortality than young adults.

Assessment of the role of gender in COVID-19 vaccine hesitancy revealed that men are more willing to accept the vaccine than women [28,29,30,38,48,49], and this held true across cultures (Arab countries [48,49], China [28], Ireland, and the UK [29]). Only one cross-sectional study in the USA reported lower acceptance among men [32]. Women were reported to have adopted more negative views about vaccination [30] while men showed a lower belief in rumors and conspiracy theories surrounding COVID-19 and higher risk perception for the disease [49,77]. However, this finding should be interpreted with caution in light of sex distribution, as sampling bias cannot be ruled out.

Similar to previous findings, the current review found variations in vaccine acceptance and uptake across different race and ethnic minorities. Blacks, Hispanics, Chinese, Asian, non-Irish, mixed, or other ethnicities were more hesitant and more likely to reject the vaccines. The literature attributed this attitude to religious and cultural beliefs, norms, and concerns [37,79]. High education level and high-income status were associated with positive attitudes toward vaccination, owing to minimal barriers related to knowledge, health literacy, and cost concerns [23,24,25,27,30,35,36,42,47,49,50,51,63,65].

In addition, other factors were included, such as health condition, people with chronic diseases, occupation status, marital status, place of residence, women being pregnant or having children, and having health insurance or not. More willingness and acceptance were reported among married individuals [7,28,52], those who reside in rural or suburban areas [29,68], being employed [27,46,50,53,73], especially in professional and managerial occupations [50,53], being at risk or belonging to a vulnerable group [34,45,65], and having insurance [30,31,36,51,68]. Knowing these factors can provide guidance for organizations and professionals on people and settings that need to be targeted to enhance vaccine acceptance and improve vaccine uptake rates [79].

More publications (36 studies) reported several individual factors influencing public attitudes towards COVID-19 vaccines. The most cited factors were beliefs [30,32,34,35,49,55,56,57,62,66,68,70], knowledge, and health literacy [23,33,34,35,39,57,65,68]. Other factors such as perceived susceptibility, threats and benefits, social, religious, and political views, previous exposure to flu vaccines, and lack of trust in the governments and companies producing the vaccines were also reported. This is consistent with findings from other reviews related to COVID-19 vaccines and other vaccines [77,78,80].

Knowledge about COVID-19 vaccines is limited as illustrated in numerous studies [23,33,34,35,39,57,65,68]. Unfavorable attitudes toward vaccination was related to misbeliefs, conspiracy beliefs, and antivaccine beliefs [35,47,49,56,57,62,66,68], inadequate knowledge and health literacy [33,57,65], lower perceived risk, threat, severity, and susceptibility [24,28,38,41,43,45,46,47,48,56], political conservatism, partisanship and engagement [29,32,54,61,66], and religious conviction [37]. Nevertheless, the factors associated with more vaccination acceptance included positive subjective norms and attitudes towards vaccination in general and COVID-19 vaccination in particular [36,56,59,66], high perceived benefits [46], self-efficacy [36], institutional and government trust [25,35,37,39,59], previous exposure to flu or other vaccines [28,30,52,56,58], and prosocial concerns [61,70]. Enhancing these factors may improve the vaccination uptake rate.

Several studies examined the role of the social network and organizational factors [24,25,28,31,37,42,43,44,64,69]. Healthcare professionals appeared to be a trusted source of information. Their recommendations [28,43,44,69] in addition to support of family and friends [31,44] play an important role in shaping perceptions and behaviors towards vaccination. Significant others were reported in the literature to influence one’s attitude and behavior. Information, acknowledgement, and recommendations from family members, friends, employers, and community members were associated with favorable attitudes and a higher uptake of vaccines [25,78,80].

On the other hand, the misinformation encountered, particularly on social media, the type and frame of massages received may influence the attitude to vaccination and intention [24,37,42,64]. Propagation of myths and conspiracy theories around vaccines and promotion of the antivaccine sentiment, combined with exposure to persuasive tactics, can convince the person that the vaccine is harmful, as indicated by Sarah Ashfield et al. [81]. Accordingly, public health organizations, healthcare professionals, and media platforms can collaborate to guarantee information accuracy, deliver health promotion programs to improve levels of health literacy to enable the target population to make an informed decision. In addition, this psychosocial environmental impact implies that strategies to overcome hesitancy can be framed within models that consider these multifaceted and multileveled factors.

Regarding vaccine characteristics, many publications included in this review focused on efficacy, safety, adverse effects or toxicity of the vaccines, and cost [28,35,40,43,45,46,47,63,65,68,72,73], which were the most significant characteristics and concerns about COVID-19 vaccines. These were also common factors highlighted in other reviews about COVID-19 vaccines and other vaccines [77,78,80].

Beyond these, the present review also found other factors that further contribute to our understanding of the barriers to vaccination uptake. Immunity duration [68], vaccination timeframe [63], fake or low-quality vaccines [37,46], beliefs about the consequences [47], country of vaccine origin [68], information about inactivated vaccines [72], and doubts about technology used in production [37,67] allow a detailed understanding of how to approach vaccine-hesitant groups to increase acceptance and uptake of the vaccines. These concerns can be addressed via awareness campaigns guided by physicians and other healthcare professionals to foster trust in health authorities, assure the public, and illustrate the role of vaccination in acquiring herd immunity and preventing disease transmission.

As illustrated above, the majority of the studies in this review addressed factors associated with vaccination attitude at the micro-meso level; however, there is a lack of publications that address the factors on the upper level of the socio-ecological model. Determinants on the macro-level of the model, including policy/regulations, broad sociocultural, religious, political, and environmental factors, and the influence they may exert on COVID-19 vaccine uptake are underexplored to date, at least in the results presented in our review. This gap in evidence necessitates further research to comprehensively tackle the issue of vaccine hesitancy. Another important output for this scoping review reflects the gap in clinical evidence concerning the COVID-19 vaccine efficacy, safety, and side effects to date, which showed an influence in shaping public hesitancy and refusal of the vaccines. Clinical research is needed to fill this gap and manage sharing evidence that will alleviate public concerns and enhance vaccine acceptance.

The included studies reported global data which can be seen as a strength of this review. On the other hand, our review has some limitations. Only the articles in English were included; this may have potentially introduced bias or resulted in missing important literature. We did not include one keyword, “Antivax,” in our search strategy; however, when we did, only one new publication was found, therefore, this would unlikely have a noticeable effect on our results. The majority of the included studies are cross-sectional, which limits the ability to infer the causation between the various factors and public attitudes towards the COVID-19 vaccination. Most of these studies used self-administered surveys that may lead to biases. Furthermore, uncontrolled health conditions of the target populations and the various global healthcare systems in the studies included in this review may have had a misleading influence on the results.

Understanding various population needs and the factors shaping public attitudes towards the vaccines would support planning for evidence-based multilevel interventions in order to enhance the vaccine uptake globally. In our findings, we were able to report factors on the individual, social, and organizational levels. Future research should focus on exploring the cultural, economic, and political factors influencing public attitudes towards the COVID-19 vaccination.

## 5. Conclusions

This review highlights the complexity of the topic. Our findings show that attitudes toward COVID-19 vaccines is shaped by factors that are multifaceted and multileveled. A combination of a set of complementary multilevel interventions and engagement of diverse players, recipients, and settings may be helpful to improve the vaccination uptake to win the fight against this pandemic.

## Figures and Tables

**Table 1:** Search strategies.

Search	Search Term
#1	“Corona” OR “SARS-CoV-2” OR “COVID 19”
#2	“Vaccine” OR “Vaccination”
#3	“Acceptance” OR “Agreement” OR “Willingness” OR “Refusal” OR “Resistance” OR “Confidence” OR “Hesitancy” OR “Antivaxx” OR “Antivaxxers” OR “Antivaccine” OR “Anti-vaccine”
#4	#1, #2, and #3

**Table 2:** Baseline characteristics of the selected studies.

First Author/Year of Publication	Study Design	Study Setting	Population	Sample Size	Mean Age (in Years)	Attitudes towards COVID-19 Vaccines
Chen, T.E. 2021 [23]	Experimental design	Online experiment	Chinese adults	413	Aged between 18 and 60 (M = 24.70, SD = 9.55)	Attitudes towards COVID-19 vaccination scores were highly favorable and the intention to get vaccinated was high
Chen, M.S. 2020 [24]	Cross-sectional	Online questionnaire	Chinese adults	3195	Majority aged 18–44	83.8% were willing to receive a COVID-19 vaccine, 13.6% were unsure
Lazarus, J.V. 2020 [25]	Cross-sectional		19 countries (global)	13,426	Majority aged 25–54	46.8% completely agreed to accept a COVID-19 vaccine
Bell, S. 2020 [26]	Cross-sectional survey	Online social media strategy	Parents and guardians (aged 16+ years) who reported living in England with a child aged 18 months or under	1190	33	Acceptability of a future COVID-19 vaccine
Coustasse, A. 2020 [63]	Review		US population			50% willing to get a vaccine in study 1 65% willing to get a vaccine in study 2
Al-Mohaithef, M. 2020 [27]	Web-based cross-sectional study	Public	General public in Saudi Arabia	992	Most of the respondents aged 26–35	Willingness to accept a vaccine
Robles, A.S. 2020 [73]	Conference paper		Nevada			Vaccine acceptance
Wang, J. 2020 [28]	Online cross-sectional survey	Public	China	2058	Adults	Willingness to accept a vaccine
Lin, C. 2021 [68]	Systematic review			126 surveys		Vaccine intention and acceptance
Murphy, J. 2021 [29]	Cross-sectional survey	Online survey	General adult populations of Ireland and the United Kingdom	3066	Mostly 55–64 (Ireland) and 45–54 (UK)	COVID-19 vaccine hesitancy and resistance, including psychological indicators
Akarsu, B. 2020 [30]	Cross-sectional web-based survey	Public	General public in Turkey	759	32.41 ± 9.92	Vaccination acceptance
Seale, H. 2021 [31]	National cross-sectional survey	Online survey	Australian adults (18 years and older)	1420	Mostly 30–49	Perceptions and behaviors towards a future COVID-19 vaccine
Hursh, S.R. 2020 [32]	Cross-sectional survey	Online using crowdsourcing platform Amazon Mechanical Turk (mTurk)	Participants from the United States	534	41.9	Evaluation of COVID-19 vaccine demand
Biasio, L.R. 2020 [33]	Online survey	Online study	Italian adults	885	Majority aged 31–50	Perceptions about getting a vaccine
Kourlaba, G. 2021 [34]	Cross-sectional survey	Computer-assisted telephone interviewing (CATI) and computer-assisted web interviewing (CAWI)	Adult Greeks	1004	41.7 (17.7)	Willingness to receive a COVID-19 vaccine
Fisher, K. 2020 [35]	Cross-sectional survey	Internet survey and telephone interviews	US general public	1000	Adults	Vaccine hesitancy
Guidry, J.P.D. 2021 [36]	Cross-sectional survey	Online survey	US adults	788	45.9	Willingness to get a COVID-19 vaccine
Jung, H. 2020 [70]	Longitudinal survey, two experiments		United states	Survey: 2490 Experiment: 800		Vaccination intention
Popa, G.L. 2020 [37]	Face-to-face cross-sectional survey	Public	Romanian respondents	1647	Median age, 37	Vaccination acceptance
Detoc, M. 2020 [38]	Cross-sectional survey	Online via social networks	General population in France	3250	Mostly 30–49	Intention to get vaccinated against COVID-19
Prati, G. 2021 [39]	Online survey	Online study	General public in Italy	624	Between 18 and 72 years	Willingness to accept a vaccine
Marco-Franco, J.L. 2021 [40]	Cross-sectional, legal analysis		Several countries			Vaccine hesitancy
Caserotti, M. 2021 [41]	Cross-sectional online questionnaire (pre-lockdown, during lockdown, and post-lockdown) Note: “different participants every phase”	Various institutional and personal social channels related to the research team	Italian residents	2267	25–65	The percentage of people who accepted a vaccine was high during the lockdown
Bogart, L.M. 2021 [69]	RCT	Community-based	Black Americans	101	50.3 (11.5)	COVID-19 mistrust beliefs, COVID-19 vaccine or treatment hesitancy
Alley, S.J. 2021 [42]	Repeated cross-sectional survey	Online	Australian adults	575	Mostly over 55 years	Willingness to get vaccinated against COVID-19
Puri, N. 2020 [64]	Review	Social media				Vaccine hesitancy
Reiter, P.L. 2020 [43]	Online survey	Online study	US general public	2006	>18	Willingness to accept a vaccine
Feleszko, W. 2021 [44]	Poland, an online omnibus survey tool	Public	Poland public	1066	18–65	37% supported COVID-19 vaccination
Danchin, M. 2020 [65]	Review					Vaccine refusal
Harapan, H. 2020 [45]	Cross-sectional online survey	Online study	General population in Indonesia	1359	More than half of the respondents were aged 21–30 years	50 or 95% effective COVID-19 vaccine
Lin, Y. 2020 [46]	Nationwide cross-sectional self-administered online survey	Public	China	3541	Adults	83.5% said yes to the intent to get a vaccine
Williams, L. 2020 [47]	Cross-sectional study with open-ended questions about the factors	Online	Older adults and people with respiratory chronic diseases	527	Older sample with adults aged 65	86% of respondents wanted to receive a COVID-19 vaccine
Yin, F. 2021 [72]	Media analysis through a proposed novel behavioral dynamics model, SRS/I (susceptible–reading–susceptible/immune)	Microblogging platform Weibo (social media)	Chinese citizens (living in China and abroad)	1.75 million Weibo messages		COVID-19 vaccine acceptance
Alqudeimat, Y.2021 [48]	Web-based cross-sectional study	Public (adults living in Kuwait)	Kuwait	2368	>21	Participants were willing to accept a COVID-19 vaccine once available (53.1%)
Sallam, M. 2021 [49]	Cross-sectional online survey	Online study	Jordan, Kuwait, and Saudi Arabia	3414		Vaccine acceptance (29.4%)
Wong, L.P. 2020 [50]	Cross-sectional online survey	Online survey	Malaysian general public	1159	Adults	Willingness to accept a vaccine
Nguyen, K.H. 2020 [51]	CDC-conducted household panel surveys	Internet survey	US general public		Adults	Willingness to accept a vaccine
Wang, K.L. 2021 [52]	Cross-sectional	Online questionnaire	Working population in Hong Kong (HK), China	1196	Majority aged 40–49	Standardized rate of vaccine acceptance in the first survey was 44.2% and 34.8% in the second survey
Largent, E.A. 2020 [71]	Gallup panel	Online study	US adults	2730	Not available	Acceptability of COVID-19 vaccines
LaVecchia, K. 2020 [53]	National cross-sectional survey	Online using computer- assisted web interviews (CAWI)	Italians aged 15–85 years	1055	33.2	Attitudes towards a potential COVID-19 vaccine
Ward, J.K. 2020 [54]	Cross-sectional survey	Online	French population of 18 years of age	5018	Mostly less than 35	Attitudes to a future COVID-19 vaccine
Romer, D. 2020 [55]	Two-wave follow-up surveys	Online using Qualtrics	US general population	1050	Mostly 60+	Intention to get vaccinated against COVID-19
Sherman, S.M. 2020 [56]	Cross-sectional online survey	Online study	UK adults	1500	46 ± 15.8	Vaccination intention
McCaffery, K.J. 2020 [57]	National cross-sectional community online survey		Australian general public, adults aged over 18 years	4362	42.6 ± 17.4	Vaccine hesitancy
Pogue, K. 2020 [58]	Cross-sectional survey	Public online survey	US respondents	316		COVID-19 vaccine intention
Taylor, S. 2020 [59]	Cross-sectional survey	Public Internet-based study	American and Canadian adults	3674	53 ± 15	Vaccination hesitancy
Reuben, R.C. 2020 [60]	Cross-sectional online survey with a semi-structured questionnaire using a snowball sampling technique	Public	North-central Nigeria	589	80.6% were 18–39 years	29% accepted to take a vaccine
Corpuz, R. 2020 [61]	Cross-sectional Amazon’s Mechanical Turk (MTurk), an online survey tool	Online study	US general public	209	Mean age M = 33.4 years (SD = 11.4)	Endorsement of the vaccines
Bertin, P. 2020 [62]	Two cross-sectional studies. An online questionnaire was disseminated by the authors on Facebook, Twitter, and Linkedin	Online studies	General public in France	396	M_age_ = 26.1, SD = 10.3, min = 18, max = 70	Negatively predicted participants intention to get the vaccine
Ling, R. 2020 [66]	Review	Social media				Anti-vaccination
Dube, E. 2020 [67]	Review					Vaccine hesitancy

**Table 3:** Sociodemographic characteristics associated with hesitancy or acceptance of COVID-19 vaccines.

First Author	Hesitancy (−) or Acceptance (+) of COVID-19 Vaccines	Sociodemographics Shaping Public Attitudes towards COVID-19 Vaccines
Chen, T.E. 2021 [23]	+	- High education level - Increasing age
Chen, M.S. 2020 [24]	+	- Male gender - High income - Education level - Han nationality
Lazarus, J.V. 2020 [25]	+	- Age older than 25 - Male gender - High income - High education level
	−	- Sick people or sick family members
Bell, S. 2020 [26]	−	- Black, Asian, Chinese, mixed, or other ethnicity - Low income
Coustasse, A. 2020 [63]	+	- 60 years and older - Non-Hispanic Whites - High education level - High income
Al-Mohaithef, M. 2020 [27]	−	- Older age - Being married - High education level - Non-Saudi - Employed in the government sector
Robles, A.S. 2020 [73]	+	- Age - Ethnicity - Chronic disease - Education level - Employment status - Country
Wang, J. 2020 [28]	+	- Male gender - Being married
Lin, C. 2021 [68]	+	- College degrees - Income, - Insurance - Living in rural or larger areas - Gender - Race
Murphy, J. 2021 [29]	−	Irish sample, vaccine-hesitant - Female gender - Aged between 35 and 44 years - No mental health problem Irish sample, vaccine-resistant - Aged 35–44 years - Residing in a city - Non-Irish ethnicity - Lower income - Underlying health condition UK sample, vaccine-hesitant - Female gender - Younger than 65 UK sample, vaccine-resistant - Younger age - More likely to reside in a suburb - In the three lowest income brackets - Being pregnant
Akarsu, B. 2020 [30]	−	- Female gender - Unemployed
	+	- Have SSI or private health insurance - Have children - Those who were thinking about getting their child a COVID-19 vaccine were more willing to get vaccinated - High level of education
Seale, H. 2021 [31]	+	- Female gender - Aged 70 years and above - Reporting chronic disease - Holding private health insurance
Hursh, S.R. 2020 [32]	−	- Male gender
Kourlaba, G. 2021 [34]	+	- Aged > 65 years old - Those belonging to vulnerable groups or members of their household belonging to vulnerable groups
Fisher, K. 2020 [35]	-	- Young age - Black race - Low educational attainment
Guidry, J.P.D. 2021 [36]	+	- Education - Having insurance - Age - Race/ethnicity
Detoc, M. 2020 [38]	+	- Older age, - Male gender
Alley, S.J. 2021 [42]	−	- Low education - Female gender
Danchin, M. 2020 [65]	−	- Low education - Low income - Potentially more prone to infectious diseases - Women aged < 35 years - People aged > 75 years who are at a higher risk of disease from COVID-19
Lin, Y. 2020 [46]	+	- Self-employed and service sector workers
Williams, L. 2020 [47]	+	- White ethnicity - High education level - High income - High-risk/shielding
Alqudeimat, Y. 2021 [48]	+	- Male gender
Sallam, M. 2021 [49]	+	- Male gender - High education levels - History of chronic disease
Wong, L.P. 2020 [50]	+	- Higher education levels - Professional and managerial occupations - High income
Nguyen, K.H. 2020 [51]	−	- Young adults - Female gender - Non-Hispanic Black (Black) persons - Adults living in nonmetropolitan areas - Adults with lower educational attainment - Low income - No health insurance
Wang, K.L. 2021 [52]	+	- Young age - Male gender - Being married
Largent, E.A. 2020 [71]	+	- Non-Black respondents more likely to get vaccinated - Respondents with a bachelor’s degree or higher
LaVecchia, K. 2020 [53]	+	- Age above 55 - Professionals, managers, teachers, and manual workers

**Table 4:** Individual factors associated with hesitancy or acceptance of COVID-19 vaccines.

First Author	Hesitancy (−) or Acceptance (+) of COVID-19 Vaccines	Individual Factors Shaping Public Attitudes towards COVID-19 Vaccines
Chen, M.S. 2020 [24]	+	- Confidence, satisfaction, and worries about risks - Attention to relevant COVID-19 information - Perceived views of the severity of COVID-19 disease - Degree of concern regarding the COVID-19 pandemic
Lazarus, J.V. 2020 [25]	+	- Trust in the government
Wang, J. 2020 [28]	+	- Perceiving a high risk of infection - Being vaccinated against influenza in the previous season
Lin, C. 2021 [68]	−	- Belief that vaccines are unnecessary - Inadequate information - General anti-vaccine stand - Willingness to pay
Murphy, J. 2021 [29]	−	- Irish sample: more likely to have voted for the political party Sinn Féin or an independent politician in the previous general election
Akarsu, B. 2020 [30]	+	- Got a seasonal flu vaccine - High level of anxiety
	−	- “Afraid of the side effects of the vaccines” - “Do not think it can be reliable as it will be a new vaccine” - “COVID-19 infection is a biological weapon” - “The vaccine will serve those who produce this virus”
Hursh, S.R. 2020 [32]	−	- Greater conspiracy beliefs and political conservatism
Biasio, L.R. 2020 [33]	Attitudes to a COVID-19 vaccine	- Health literacy
Kourlaba, G. 2021 [34]	+	- Those believing that the COVID-19 virus was not developed in laboratories by humans - Those believing that coronavirus is far more contagious and lethal compared to the H1N1 virus - Those believing that next waves are coming - Higher knowledge score regarding symptoms, transmission routes, and prevention and control measures against COVID-19
Fisher, K.A. 2020 [35]	−	- Vaccine-specific concerns - Need for more information - General anti-vaccine beliefs - Lack of trust
Guidry, J.P.D. 2021 [36]	+	- Positive subjective norms - A positive attitude toward vaccines in general - Perceived susceptibility to COVID-19 - High perceived benefits of the vaccines - Scoring low on barriers to the vaccines - Scoring high on self-efficacy - High perceived behavioral control
Jung, H. 2020 [70]	+	- Prosocial concern for vaccination motivates vaccination in more and less populated regions
Popa, G.L. 2020 [37]	−	- Lack of information - Fear of adverse reactions - Fears of toxicity and poor quality related to vaccine components - Doubts about the technology used to produce the vaccines - Personal reasons to refuse vaccines (which included religious conviction) - Lack of trust in the healthcare system
Detoc, M. 2020 [38]	+	- Fear about COVID-19 and individual perceived risk
Prati, G. 2021 [39]	Attitudes to a COVID-19 vaccine	- Being worried about the non-natural origin of the virus and the role of the institutional trust
Caserotti, M. 2021 [41]	+	- Likelihood of getting the infection - Perceived severity of the disease
Reiter, P.L. 2020 [43]	+	- Likelihood of getting the COVID-19 infection in the future - Perceived severity of the COVID-19 infection
Danchin, M. 2020 [65]	+	- Adequate health literacy
Harapan, H. 2020 [45]	+	- Perceived risk of the COVID-19 infection
Lin, Y. 2020 [46]	+	- Perceiving overall health as very good - Perceiving the benefit of feeling less worried of contracting coronavirus after getting a vaccine - Perceiving the benefit of a COVID-19 vaccine in reducing the risk of infection and resultant complications - If given adequate information and if taken by many in the general public
Williams, L. 2020 [47]	Attitudes to a COVID-19 vaccine	- The perception that COVID-19 will persist over time - Perceiving the media to have overexaggerated the risk - The “beliefs about consequences” TDF domain, with themes relating to personal health, health consequences to others, and severity of COVID-19
Alqudeimat, Y. 2021 [48]	−	- Likelihood of infection - Viewed vaccines in general to have health-related risks
Sallam, M. 2021 [49]	−	- Beliefs that COVID-19 vaccines are intended to inject microchips into recipients and that the vaccines are related to infertility
Wang, K.L. 2021 [52]	+	- Influenza vaccine uptake during the previous year
Largent, E.A. 2020 [71]	−	- Republicans and Independents were less likely to get vaccinated than Democrats
Ward, J.K. 2020 [54]	Attitudes to a COVID-19 vaccine	- Political partisanship and engagement with the political system
Romer, D. 2020 [55]	−	- Belief in three COVID-19-related conspiracy theories
Sherman, S.M. 2020 [56]	+	- Having been vaccinated for influenza the previous winter - Perceiving a great risk of COVID-19 - Positive general COVID-19 vaccination beliefs and attitudes - Weak beliefs that the vaccination would cause side effects - Greater perceived information sufficiency to make an informed decision about COVID-19 vaccination - Lower endorsement of the notion that only people who are at risk of serious illness should be vaccinated for COVID-19
McCaffery, K.J. 2020 [57]	−	- Beliefs and misinformation about COVID-19/vaccines - Inadequate health literacy
Pogue, K. 2020 [58]	+	- Respondents who routinely got vaccines were more likely to be receptive to receiving a COVID-19 vaccine - The greater the perceived impact of COVID-19 in America, the more receptive the respondent was to receive a potential COVID-19 vaccine
Taylor, S. 2020 [59]	−	- Mistrust of vaccine benefits - Worries about unforeseen future negative effects - Concerns about commercial profiteering - Preference for natural immunity
Reuben, R.C. 2020 [60]	−	- No confidence in the present intervention by Chinese doctors
Corpuz, R. 2020 [61]	+	- Those exhibiting a slow life history orientation were more likely to endorse mandatory vaccination for COVID-19
	−	- Social and political conservatism
Bertin, P. 2020 [62]	−	- COVID-19 conspiracy beliefs
Ling, R. 2020 [66]	−	- Confirmation bias, consumption of only those news that confirm the pre-existing attitudes and beliefs

**Table 5:** Social and organizational factors associated with hesitancy or acceptance of COVID-19 vaccines.

First Author	Hesitancy (−) or Acceptance (+) of COVID-19 Vaccines	Social Networks and Organizational Factors (Family, Friends, HC Providers, Employers) and Media Shaping Public Attitudes towards COVID-19 Vaccines
Chen, T.E. 2021 [23]	+	- Type of messages received and message frames - Outcome uncertainty - Number format - Numeracy skills
Lazarus, J.V. 2020 [25]	+	- Accepting their employer’s recommendation to do so
Wang, J. 2020 [28]	+	- Valuing their doctor’s recommendations
Seale, H. 2021 [31]	+/−	- Decision to get vaccinated would be supported by family and friends
Popa, G.L. 2020 [37]	−	- Disinformation (through classic media, social media, and the Internet)
Bogart, L.M. 2021 [69]	+	- Social service and healthcare providers
Alley, S.J. 2021 [42]	−	- Infrequent users of traditional media
Puri, N. 2020 [64]	−	- Anti-vaccination messages on social platforms
Reiter, P.L. 2020 [43]	+	- Healthcare providers recommending vaccination
Feleszko, W. 2021 [44]	+	- Recommended by a family doctor - Someone of family members/friends was vaccinated - Need a vaccination certificate to enter some countries

**Table 6:** Characteristics of COVID-19 vaccines associated with hesitancy or acceptance of the vaccines.

First Author	Hesitancy (−) or Acceptance (+) of COVID-19 Vaccines	Characteristics of COVID-19 Vaccines Shaping Public Attitudes towards COVID-19 Vaccines
Chen, M.2020 [24]	+	- If the vaccine is domestic, not imported
Lazarus, J.V. 2020 [25]	+	- Vaccine proved safe and effective by the government
Coustasse, A. 2020 [63]	+/−	- Effectiveness estimate of the vaccine - Safety based on newness and adverse effects - Lack of testing - Vaccination timeframe - Who will have access to it - Cost to consumers - How states and the federal government will determine vaccination methods - Getting COVID-19 from the shot - Fear of side effects from an untested vaccine
Robles, A.S. 2020 [73]	+/−	- Perception of efficacy, safety, and adverse effects of the vaccines - Source of information - Conspiracy theories - Reactance and outrage regarding new information
Wang, J. 2020 [28]	+/−	- Efficacy of COVID-19 vaccination - Concerns about vaccine safety
Lin, C. 2021 [68]	+/−	- Newness of COVID-19 vaccines - Inadequate information - Unknown/short duration of immunity - Cost - Country of vaccine origin - Fear of side effects, safety, and effectiveness
Fisher, K.A. 2020 [35]	−	- Vaccine-specific concerns - Need for more information
Popa, G.L.P. 2020 [37]	−	- Fear of adverse reactions - Fear of toxicity and poor quality related to vaccine components - Doubts about the technology used to produce the vaccines - Price
Marco-Franco, J.E. 2021 [40]	−	- Worries about the side effects, safety and effectiveness of the vaccines
Reiter, P.L. 2020 [43]	+	- Effectiveness of a COVID-19 vaccine
Danchin, M. 2020 [65]	+	- Vaccine safety and effectiveness
Harapan, H. 2020 [45]	+	- The baseline effectiveness of the vaccines
Lin, Y. 2020 [46]	+/−	- Concerns about faulty/fake vaccines - Affordability and high price - Safety and efficacy - Confidence and preference of domestically made vaccines
Williams, L. 2020 [47]	+/−	- Personal concerns regarding vaccine safety
Yin, F. 2021 [72]	+	- The majority thought the price was low - Positive views on side effects - Information about inactivated vaccines (inactivated vaccines are more accepted)
Wang, K.L. 2021 [52]	−	- Doubts of effectiveness - Thought of the vaccines as unnecessary - More accepted in the first wave compared to the third wave
Dube, E. 2020 [67]	−	- Vaccine development is being pushed - COVID-19 vaccine antigen-carrying platforms have never been used - The production of new COVID-19 vaccines will not meet demand - Conspiracy theories - More than one type of COVID-19 vaccines is likely to be used within a country. Thus, the safety and efficacy profiles may vary

## Data Availability

The data presented in this study are available in Supplementary materials.

## Supplementary Materials

The following are available online at https://www.mdpi.com/article/10.3390/vaccines9060548/s1, Table S1. Summary table of the factors associated with hesitancy or acceptance of COVID-19 vaccines.

## Appendix A

**Table A1:** Preferred Reporting Items for Systematic Reviews and Meta-Analyses extension for Scoping Reviews (PRISMA-ScR) Checklist.

Section	Item	Prisma-ScR Checklist ITEM	Reported on Page #
Title			
Title	1	Identify the report as a scoping review.	1
Abstract			
Structured summary	2	Provide a structured summary that includes (as applicable): background, objectives, eligibility criteria, sources of evidence, charting methods, results, and conclusions that relate to the review questions and objectives.	1
Introduction			
Rationale	3	Describe the rationale for the review in the context of what is already known. Explain why the review questions/objectives lend themselves to a scoping review approach.	2
Objectives	4	Provide an explicit statement of the questions and objectives being addressed with reference to their key elements (e.g., population or participants, concepts, and context) or other relevant key elements used to conceptualize the review questions and/or objectives.	3
Methods			
Protocol and registration	5	Indicate whether a review protocol exists; state if and where it can be accessed (e.g., a Web address); and if available, provide registration information, including the registration number.	3
Eligibility criteria	6	Specify characteristics of the sources of evidence used as eligibility criteria (e.g., years considered, language, and publication status), and provide a rationale.	3
Information sources *	7	Describe all information sources in the search (e.g., databases with dates of coverage and contact with authors to identify additional sources), as well as the date the most recent search was executed.	3
Search	8	Present the full electronic search strategy for at least 1 database, including any limits used, such that it could be repeated.	3
Selection of sources of evidence †	9	State the process for selecting sources of evidence (i.e., screening and eligibility) included in the scoping review.	3
Data charting process ‡	10	Describe the methods of charting data from the included sources of evidence (e.g., calibrated forms or forms that have been tested by the team before their use, and whether data charting was done independently or in duplicate) and any processes for obtaining and confirming data from investigators.	4
Data items	11	List and define all variables for which data were sought and any assumptions and simplifications made.	4
Critical appraisal of individual sources of evidence §	12	If done, provide a rationale for conducting a critical appraisal of included sources of evidence; describe the methods used and how this information was used in any data synthesis (if appropriate).	NA
Synthesis of results	13	Describe the methods of handling and summarizing the data that were charted.	4
Results			
Selection of sources of evidence	14	Give numbers of sources of evidence screened, assessed for eligibility, and included in the review, with reasons for exclusions at each stage, ideally using a flow diagram.	5
Characteristics of sources of evidence	15	For each source of evidence, present characteristics for which data were charted and provide the citations.	6
Critical appraisal within sources of evidence	16	If done, present data on critical appraisal of included sources of evidence (see item 12).	NA
Results of individual sources of evidence	17	For each included source of evidence, present the relevant data that were charted that relate to the review questions and objectives.	8
Synthesis of results	18	Summarize and/or present the charting results as they relate to the review questions and objectives.	13
Discussion			
Summary of evidence	19	Summarize the main results (including an overview of concepts, themes, and types of evidence available), link to the review questions and objectives, and consider the relevance to key groups.	28
Limitations	20	Discuss the limitations of the scoping review process.	30
Conclusions	21	Provide a general interpretation of the results with respect to the review questions and objectives, as well as potential implications and/or next steps.	30
Funding			
Funding	22	Describe sources of funding for the included sources of evidence, as well as sources of funding for the scoping review. Describe the role of the funders of the scoping review.	31

JBI = Joanna Briggs Institute; PRISMA-ScR = Preferred Reporting Items for Systematic Reviews and Meta-Analyses extension for Scoping Reviews. * Where sources of evidence (see second footnote) are compiled from, such as bibliographic databases, social media platforms, and Web sites. † A more inclusive/heterogeneous term used to account for the different types of evidence or data sources (e.g., quantitative and/or qualitative research, expert opinion, and policy documents) that may be eligible in a scoping review as opposed to only studies. This is not to be confused with information sources (see first footnote). ‡ The frameworks by Arksey and O’Malley (6) and Levac and colleagues (7) and the JBI guidance (4, 5) refer to the process of data extraction in a scoping review as data charting. § The process of systematically examining research evidence to assess its validity, results, and relevance before using it to inform a decision. This term is used for items 12 and 19 instead of “risk of bias” (which is more applicable to systematic reviews of interventions) to include and acknowledge the various sources of evidence that may be used in a scoping review (e.g., quantitative and/or qualitative research, expert opinion, and policy document).
