# Supplementary material for: Factors Influencing Public Attitudes towards COVID-19 Vaccination: A Scoping Review Informed by the Socio-Ecological Model
Source: Vaccines (Basel). 2021 May 24;9(6):548. doi: 10.3390/vaccines9060548 (PMC8225013; doi:10.3390/vaccines9060548)
Supplement: Supplementary file 1 [file vaccines-09-00548-s001.zip › vaccines-1207466-supplementary.pdf]

Table S1. Summary table of the factors associated with hesitancy or acceptance of COVID-19 vaccines.

First Author/ Year of Publication	Hesitancy (-) or Acceptance (+) of COVID-19 vaccine	Socio-demographics Shaping public Attitude towards COVID-19 Vaccine	Individual Factors Shaping Public Attitude Towards COVID-19 Vaccine	Social Networks and Organizational Factors (Family, Friends, HC Providers, Employers) and Media Shaping Public Attitude towards COVID-19 Vaccine	Characteristics of COVID-19 Vaccine Shaping Public Attitude towards COVID-19 Vaccine
Chen, T.E. 2021 [23]	+	-High education level<br>-Increasing age		-Type of messages received, and message frames<br>-Outcome uncertainty<br>-Number format<br>-Numeracy skills	
Chen, M.S. 2020 [24]	+	-Male gender<br>-High income<br>-Education level<br>-The Han nationality	-Confidence, satisfaction and worry about risks.<br>-Attention to relevant COVID-19 information<br>-Perceived views of the severity of COVID-19 disease<br>-Degree of concern regarding the COVID-19 pandemic		-If domestic vaccine and not imported vaccine
Lazarus, J. V. 2020 [25]	+<br>-	-Age older than 25<br>-Male gender<br>-High income<br>-High education level<br>-Sick people or sick family members	-Trust in government.	-Accept their employer's recommendation to do so	-Vaccine approved safe and effective by the government
Bell, S. 2020 [26]	-	-Black, Asian, Chinese, Mixed or other ethnicity<br>-Low income			
Coustasse, A. 2020 [27]	+	-60 years and older<br>-Non-Hispanics Whites			-Effectiveness estimate of the vaccine

	+/-	-High education level<br>-High income			-Safety based on its newness and adverse effects<br>-Lack of testing<br>-Time frame for a vaccine<br>-Who will have access to it<br>-Cost to consumers,<br>-How states and the federal government will determine vaccination methods<br>-Catching COVID-19 from the shot<br>-Fear side effects from an untested vaccine
Al-Mohaithef, M. 2020 [28]	-	-Older age<br>-Being married<br>-High education level<br>-Non-Saudi<br>-Employed in government sector			
Robles, AS. 2020 [29]	+<br>+/-	-Age,<br>-Ethnicity<br>-Chronic disease<br>-Education level<br>-Employment status<br>-Country			-Perception of efficacy, safety, and adverse effects of vaccine,<br>-Source of information<br>-Conspiracy theory<br>-Reactance and outrage to new information.
Wang, J. 2020 [30]	+<br>+/-	-Male gender<br>-Being married	-Perceiving a high risk of infection<br>-Being vaccinated against influenza in the past season	-Value doctor's recommendations	-The efficacy of COVID-19 vaccination<br>-Concerns about vaccine safety

Lin, C. 2021 [31]	+<br>-	-College degrees<br>-Income,<br>-Insurance,<br>-Living in rural or larger areas<br>-Gender<br>-Race.	-Belief that vaccines are unnecessary<br>-Inadequate information,<br>-General anti-vaccine stand<br>-Willingness to pay.		-Newness of COVID-19 vaccines<br>-Inadequate information<br>-Unknown/short duration of immunity<br>-Cost<br>-Country of vaccine origin<br>-Fear of side effects, safety, and effectiveness
Murphy, J. 2021 [32]	-	**Irish sample vaccine hesitant**<br>-Female gender<br>-Aged between 35 and 44 years,<br>-Have no mental health problem.<br><br>**Irish sample vaccine resistant**<br>-Aged 35–44 years<br>-Residing in a city<br>-Non-Irish ethnicity<br>-Lower income<br>-Have an underlying health condition<br><br>**UK sample vaccine hesitant**<br>-Female gender<br>-Younger than 65<br><br>**UK sample vaccine resistant**<br>-Age younger<br>-More likely to reside in a suburb<br>-In the three lowest income brackets<br>-Being pregnant	-Irish sample, more likely to have voted for the political party Sinn Féin or an independent political in the previous general election.		
Akarsu, B. 2020 [33]	-	-Female gender<br>-Unemployed	**-**“Afraid of the side effects of vaccine”		

	+	-Have SSI or private health insurance<br>-Have children<br>-Those who were thinking about getting their child COVID-19 vaccine were more willing to get vaccinated.<br>-High level of education	-“Don't think it can be reliable as it will be a new vaccine”<br>-“COVID-19 infection is a biological weapon<br>-“The vaccine will serve those who produce this virus”<br>-Got seasonal flu vaccine<br>-High level of anxiety
Seale, H. 2021 [34]	+	-Female gender.<br>-Ged 70 years and above<br>-Reported chronic disease<br>-Held private health insurance.	-Decision to vaccinate would be supported by family and friends.
Hursh, S.R. 2020 [35]	-	-Male gender	-Greater conspiracy beliefs and political conservatism
Biasio, L.R. 2020 [36]	Attitudes to a COVID-19 vaccine		-Health literacy
Kourlaba, G. 2021 [37]	+	-Aged > 65 years old<br>-Those who either they or a member of their household belonged to a vulnerable group.	-Those believing that the COVID-19 virus was not developed in laboratories by humans<br>-Those believing that coronavirus is far more contagious and lethal compared to the H1N1 virus<br>-Those believing that next waves are coming<br>-Higher knowledge score re-

			garding symptoms, transmission routes and prevention and control measures against COVID-19.		
Fisher, K. 2020 [38]	-	-Young age<br>-Black race<br>-Low educational attainment.	-Vaccine-specific concerns<br>-A need for more information<br>-General anti-vaccine beliefs<br>-A lack of trust.		-Vaccine-specific concerns<br>-A need for more information.
Guidry, J. P.D. 2021 [39]	+	-Education<br>-Having insurance<br>-Age<br>-Race/ethnicity	-Positive subjective norms<br>-A positive attitude toward the vaccines in general<br>-Perceived susceptibility to COVID-19<br>-High perceived benefits of the vaccine<br>-Scoring low on barriers to the vaccine<br>-Scoring high on self-efficacy<br>- High perceived behavioral control.		
Jung, H. 2020 [40]	+		Prosocial concern for vaccination motivates vaccination in more and less populated regions		
Popa, G.L. 2020 [41]	-		-Lack of information<br>-Fear of adverse reactions<br>-Fears of toxicity and poor quality related to vaccine components<br>-Doubts about the technology used to produce the vaccine.<br>-Personal reasons to refuse vaccines (which included religious conviction)	-Disinformation (through classic media, social media and the Internet)	-Fear of adverse reactions<br>-Fear of toxicity and poor quality related to vaccine components<br>-Doubts about the technology used to produce the vaccine<br>-Price.

			-Lack of trust in the healthcare system.		
Detoc, M. 2020 [42]	+ -	-Older age, -Male gender	Fear about COVID-19, and individual perceived risk		
Prati, G. 2021 [43]	Attitudes to a COVID-19 vaccine		-Being worried about the non-natural origin of the virus, and the role of institutional trust		
Marco-Franco, J. E. 2021[44]	-				Worry about the side effects, safety and effectiveness of vaccine.
Caserotti, M. 2021 [45]	+		-Likelihood of getting the infection -Perceived severity for the disease.		
Bogart, L.M. 2021 [46]	+			-Social service and health care providers	
Alley, S.J. 2021 [47]	-	-Low education -Female gender.		-Infrequent users of traditional media	
Puri, N. 2020 [48]	-			-Anti vaccination messages on social platforms.	
Reiter, P.L. 2020 [49]	+		-Likelihood getting a COVID-19 infection in the future, -Perceived severity of COVID-19 infection	-Healthcare provider would recommend vaccination	-Effectiveness of a COVID-19 vaccine
Feleszko, W. 2021 [50]	+			-Recommended by a family doctor -Someone from /family/friends was vaccinated	

				-Need a vaccination certificate to enter some countries	
Danchin, M. 2020 [51]	-<br>+	-Low education<br>-Low income<br>-Potentially more prone to infectious diseases<br>-Women aged <35 years<br>-People aged >75 years, who are at higher risk of disease from COVID-19.	-Adequate health literacy.		-Vaccine safety and effectiveness.
Harapan, H. 2020 [52]	+		- Perceived risk of COVID-19 infection		-The baseline effectiveness of the vaccine.
Lin, Y. 2020 [53]	+	-Self-employed and in a service occupation	-Perceived overall health as very good<br>-Perceived the benefit of feeling less worry of contracting the coronavirus after getting the vaccine,<br>-Perceived the benefit of the COVID-19 vaccine in reducing the risk of infection and resultant complications.<br>-If given adequate information and if taken by many in the public.		-Concerns about faulty/fake vaccine,<br>-Affordability and high price,<br>-Safety and efficacy.<br>-Confidence and preference of domestically-made vaccines
Williams, L. 2020 [54]	+	-White ethnicity<br>-High education level<br>-High income<br>-High-risk/shielding	COVID-19 will persist over time,<br>-Perceiving the media to have over-exaggerated the risk.<br>-The 'beliefs about consequences' TDF domain, with		-Personal concerns of vaccine safety.

	Attitudes to a COVID-19 vaccine		themes relating to personal health, health consequences to others, and severity of COVID-19.		
Yin, F. 2021 [55]	+				-Majority thought price inexpensive<br>-Positve views on side effects<br>-nformation about inactivated vaccines (inactivated vaccines are more accepted)
Alqudeimat, Y. 2021 [56]	+<br>-	-Male gender	-Likelihood of infection,<br>-Viewed vaccines in general to have health-related risks		
Sallam, M. 2021 [57]	+<br>-	-Male gender<br>-High education levels,<br>-History of chronic disease	-Beliefs that COVID-19 vaccines are intended to inject microchips into recipients and that the vaccines are related to infertility		
Wong, L.P. 2020 [58]	+	-Higher education levels<br>-Professional and managerial occupations<br>-High income			
Nguyen, K.H. 2020 [59]	-	-Young adults<br>-Female gender<br>-Non-Hispanic Black (Black) persons			

		-Adults living in nonmetropolitan areas,<br>-Adults with lower educational attainment,<br>-Low income<br>-No health insurance			
Wang, K.L. 2021 [60]	+<br>-	-Young age<br>-Male gender<br>-Being married	-Influenza vaccine uptake last year		effectiveness<br>-Thought of vaccine as unnecessary<br>-More accepted in the first wave compared to third wave
Largent, E.A. 2020 [61]	+<br>-	-Non-Black respondents to get vaccinated, 40.9% of respondents found state<br>-Respondents with a bachelor's degree or higher	-Republicans and Independents were less likely to get vaccinated than Democrats.		
LaVecchia, K. 2020 [62]	+		-Age above 55.<br>-Workers, professionals, managers, teachers and manual workers.		
Ward, J.K. 2020 [63]	Attitudes to a COVID-19 vaccine		-Political partisanship and engagement with the political system		
Romer, D. 2020 [64]	-		-Belief in three COVID-19-related conspiracy theories.		

Sherman, S.M. 2020 [65]	+	-Having been vaccinated for influenza last winter<br>-Perceiving a great risk of COVID-19<br>-Positive general COVID-19 vaccination beliefs and attitudes<br>-Weak beliefs that the vaccination would cause side effects<br>-Greater perceived information sufficiency to make an informed decision about COVID-19 vaccination.<br>-Lower endorsement of the notion that only people who are at risk of serious illness should be vaccinated for COVID-19
McCaffery, K.J. 2020 [66]	-	-Beliefs and misinformation about COVID-19/ vaccine,<br>-Inadequate health literacy.
Pogue, K. 2020 [67]	+	-Respondents who routinely got vaccines were more likely to be receptive to receiving the COVID-19 vaccine.<br><br>-The greater the perceived impact of COVID-19 on America, the more receptive the respondent was to receive a potential COVID-19 vaccine.

Taylor, S. 2020 [68]	-	-Mistrust of vaccine benefit<br>-Worry about unforeseen future negative effects<br>-Concerns about commercial profiteering<br>-Preference for natural immunity	
Reuben, R. C. 2020 [69]	-	No confidence in the present intervention by Chinese doctors	
Corpuz, R. 2020 [70]	+<br><br>-	-Those exhibiting a slow life history orientation were more likely to endorse mandatory vaccination for COVID-19.<br><br>-Social and political conservatism	
Bertin, P. 2020 [71]	-	-Covid-19 conspiracy beliefs	
Ling, R. 2020 [72]	-	-Confirmation bias, consume only news that confirms our pre-existing attitudes and beliefs.	
Dube, E. 2020 [73]	-		The vaccine development is being pushed<br>-COVID-19 vaccine antigen carrying platforms have never been used<br>-The production of new COVID-19 vaccines will not meet demand<br>-Conspiracy theories<br>-More than one type of COVID vaccine is likely to

	be used within a country. Thus, the safety and efficacy profiles may vary.
